# Association between cerebral microbleeds and hypertension in the Swedish general population “Good Aging in Skåne” study

**DOI:** 10.1111/jch.13606

**Published:** 2019-07-05

**Authors:** Sölve Elmståhl, Katarina Ellström, Arkadiusz Siennicki‐Lantz, Kasim Abul‐Kasim

**Affiliations:** ^1^ Department of Clinical Sciences in Malmö, Division of Geriatric Medicine, Skåne University Hospital Lund University Malmö Sweden; ^2^ Department of Clinical Sciences Lund, Division of Diagnostic Radiology, Skåne University Hospital Lund University Malmö Sweden

**Keywords:** blood pressure, cerebral microbleeds, cerebral small vessel disease, cohort, hypertension

## Abstract

Cerebral microbleeds (CMB) on MRI are frequent in healthy aging individuals but precede ischemic and hemorrhagic stroke and dementia. Different etiologies have been suggested for nonlobar CMB, which have a stronger connection to hypertension (HT) than do lobar CMB. This study aimed to investigate the prevalence of CMB and the association between nonlobar/lobar CMB and different blood pressure (BP) and HT treatment conditions in a longitudinal, population‐based cohort of the Good Aging in Skåne (GÅS) study. White matter hyperintensities (WMH), CMB, atrophies, and infarctions were identified with brain 3T MRI, and BP parameters were examined in 344 randomly selected subjects between 70 and 87 years old. CMB were observed in 26% of the whole cohort, increasing from 19% of subjects in their 70s to 30% of those over 80 years of age. Of these subjects, 38% had multiple CMB, and 59% had a lobar localization. CMB were associated with severe confluent WMH (odds ratio = 7.02; 2.16‐18.84). Increasing age, being male, and having HT, impaired cognition, or a history of angina pectoris were associated with CMB. Both lobar and nonlobar CMB were associated with HT. Nonlobar CMB were particularly associated with increased BP, pulse pressure, controlled HT, and uncontrolled HT. After controlling for sex and HT, age was no longer a risk factor for CMB *In conclusion*, sex and HT are the major risk factors for CMB, especially nonlobar CMB, which suggests stricter implementation of recommended guidelines for HT treatment in the elderly.

## 1 INTRODUCTION

Cerebral microbleeds (CMB), small perivascular hemosiderin deposits visible on T2‐weighted gradient‐recalled echo (GRE) magnetic resonance imaging (MRI),^1^ result from microangiopathic processes that have been linked to cerebral amyloid angiopathy (CAA) as well as hypertensive vasculopathies and atherosclerosis.^2^ The prevalence of CMB increases with age in otherwise healthy individuals and differs from study to study, depending on cohort age, MRI technique, and CMB definition. In a pooled dataset, Cordonnier et al^3^ found a prevalence of CMB of 5% in healthy adults, 34% in subjects who suffered from ischemic stroke (IS), and 60% in those with nontraumatic intracerebral hemorrhage (ICH). The prevalence of CMB varied from 38% in elderly subjects above 80 years old in the Rotterdam scan study^4^ to 11% in the AGES‐Reykjavik community‐based cohort with a mean age of 76 years.^5^ CMB are associated with age, hypertension (HT), male sex, low cognitive performance, gait disturbances, Alzheimer's disease and vascular dementia, and hemorrhagic stroke.^1^, ^4^, ^6^ There have been conflicting results regarding diabetes mellitus, smoking, hyperlipidemia, low total cholesterol levels, cardiac ischemia, statin use, antiplatelet use, and anticoagulant use.^3^, ^6^, ^7^

Several studies have identified differences in the underlying etiology of CMB that depend on spatial distribution. The most accepted distinction is that lobar/superficial microbleeds are more related to CAA,^1^, ^4^, ^6^, ^8^, ^9^, ^10^ while deep/infratentorial CMB are more related to hypertensive vasculopathy.^4^ Lobar CMB are overrepresented in dementia, suggesting the importance of CAA in this process.^6^ Management of the traditional vascular risk factors, especially HT, for the prevention of cerebral small vessel disease (CSVD) is recommended, although studies on the effects of antihypertensive medications on CMB are lacking.^11^

The pathophysiological mechanisms behind the development of CMB are not fully understood. The link between large vessel disease, CSVD, and CMB has yet to be fully investigated. In this study, we aimed to investigate the prevalence of CMB and the connection between deep/infratentorial and lobar CMB and different aspects of peripheral blood pressure (BP) parameters as well as HT treatment in a healthy, elderly population‐based cohort.

## 2 MATERIALS AND METHODS

### 2.1 Selection and baseline examination

The Good Aging in Skåne (GÅS) study is an ongoing national, longitudinal, population‐based aging study, part of the Swedish National study on Aging and Care (SNAC).^12^ The baseline examination was conducted with 2931 subjects in nine different age cohorts ranging from 60 to 93 years who were recruited from 2001 to 2004, and an additional 1528 subjects in the age cohorts ranging from 60 to 81 years were recruited from 2007 to 2009. A randomized selection was made from the population register of the respective age‐group, and a letter of invitation was sent out. Participation rate was between 60% and 66%. Re‐examinations were offered every third year for subjects above 78 years old and every sixth year for those between 60 and 78 years old. All examinations include the same protocol with a medical examination, a questionnaire, and cognitive and physical functioning tests. For participants unable to attend the test center, home visits were offered to reduce selection bias. In conjunction with the 4th re‐examination, 821 subjects, aged 70‐87 years, were examined with a 24‐hour BP assessment, a standardized tilting test, and an intracranial and carotid Doppler. Six hundred and fifty subjects (79%) fulfilled the extended test protocol, of whom 344 participants, with a mean age of 77 years (median: 76 years; range: 70‐87 years), were subsequently invited for a supplementary MRI scan during 2015‐2017.

The baseline characteristics included age, sex, cohabitation, education, independence in daily life, physical activity (four‐grade scale), and alcohol intake (past month and year). The history of medical diagnosis covered stroke, myocardial infarction, angina pectoris, atrial fibrillation, HT, COPD, dementia, depression, diabetes, and insomnia. All data on medications, medical diagnosis, lifestyle factors, BP assessments, and cognition were derived from the medical, cognitive and questionnaire data obtained from the fourth revisit (except for two participants), the County Patient Medical Records Registry containing outpatient and inpatient records, National Inpatient and Outpatient Registry, and Swedish National Board of Health and Welfare covering all medical inpatient and outpatient visits coded according to the International Classification of Diseases (ICD) 9 and 10 in Sweden during 1987 to December 2016. The ICD classification coverage rate from the Registry for inpatient visits was 99.95% for all inpatient visits and 99.99% for outpatient visits. All drugs were coded according to the Anatomic Therapeutic Chemical (ATC) classification system. In addition, medications were categorized separately for BO1, for CO2‐CO10, and for BP‐lowering drugs combined (CO2, CO3, CO7, CO8, CO9).

General cognitive performance was assessed by the Mini‐Mental State Examination (MMSE), which has a score range from 0 to 30,^13^ and participants were categorized into three groups: normal cognition, scores of 28‐30; intermediate cognition, scores of 25‐27; and impaired cognition, scores ≤24, see Table 1. Immediate and delayed recall was assessed by a 5‐item test in which two or more false answers were categorized as a pathological response. Participants were asked whether they had suffered from some degree or a significant loss of memory during recent years.

**Table 1:** Baseline characteristics of study participants according to age‐groups and the total cohort. The prevalence of CMB and odds ratios (ORs) for coexisting CMB is from the multivariate logistic regression analysis

	Age‐groups n (%)			Cases	Prevalence of CMB	Multivariate^a^		
	70‐74 y	75‐79 y	≥ 80 y					
	n = 128	n = 127	n = 89	n (%)	n (%)	OR	95% CI	*P*
All				344 (100%)	91 (26.5%)	1.06	0.99‐1.13	0.099
70‐74 y				128 (37.2%)	24 (18.8%)	1	‐	‐
75‐79 y				127 (36.9%)	40 (31.5%)	2.03^b^	0.85‐4.83	0.109
80‐87 y				89 (25.9%)	27 (30.3%)	1.44 b	0.53‐3.88	0.477
Female	76 (59.4%)	70 (55.1%)	51 (57.3%)	197 (57.3%)	41 (20.8%)	1	‐	‐
Male	52 (40.6%)	57 (44.9%)	38 (42.7%)	147 (42.7%)	50 (34%)	2.269^c^	1.37‐3.77	0.002
Never smoked	43 (33.6%)	48 (37.8%)	37 (41.6%)	128 (37.2)	35 (27.3%)			
Former smoker	62 (48.4%)	64 (50.4%)	47 (52.8%)	173 (50.3%)	48 (27.7%)			
Smoker	22 (17.2%)	14 (11.0%)	5 (5.6%)	41 (11.9%)	8 (19.5%)			
MMSE score								
28‐30	90 (70.3%)	81 (63.8%)	53 (59.6%)	224 (65.1%)	48 (21.4%)	1	‐	‐
25‐27	26 (20.3%)	40 (31.5%)	22 (24.7%)	88 (25.6%)	30 (34.1%)	1.90	1.10‐3.27	0.028
≤24	8 (6.3%)	6 (4.7%)	9 (10.1%)	23 (6.7%)	12 (52.2%)	4.00	1.66‐9.63	0.003

a If not noted otherwise, multivariate regression analysis with age and sex as model covariates.

b Multivariate logistic regression model: sex and hypertension as model covariates.

c Multivariate logistic regression model: age and hypertension as model covariates.

### 2.2 Blood pressure measurements

Resting BP was bilaterally assessed three times by a physician as part of the medical examination; after approximately 10 minutes of rest, with the subject in a lying position, BP was assessed using a manual, auscultatory method with an appropriately sized cuff positioned at the heart level. The highest BP value between the left and right arm was used for the analysis. A 12‐channel ECG was recorded. HT was defined according to WHO criteria: systolic BP ≥ 140 mmHg, diastolic BP ≥ 90 mmHg, or use of a BP‐lowering medication.^14^ Systolic ankle pressure was determined on the tibial posterior artery using a Doppler probe and manual BP cuff. An ankle‐brachial index (ABI) below 0.9 was categorized as impaired peripheral circulation. The highest value from the left and right brachial pressures was used. Orthostatic BP was obtained immediately and 1, 3, 5, and 10 minutes after tilting from a supine position. An orthostatic reaction was defined as an immediate systolic BP drop ≥30 mm Hg and/or a systolic or diastolic BP drop of ≥20 mm Hg or 10 mm Hg, respectively, after 1‐10 minutes.^15^

### 2.3 Blood pressure phenotypes

Blood pressure phenotypes were categorized into the five groups: (a) *Healthy controls* (normal resting BP at medical examination, no diagnosis of HT and treatment, no BP‐lowering drugs, no drugs indicated for HT); (b) *Controlled HT* (normal resting BP at medical examination, diagnosis of HT and treatment according to examination or Medical Registry, takes BP‐lowering drugs indicated for HT); (c) *Uncontrolled HT* (hypertensive resting BP at medical examination, diagnosis of HT and treatment according to examination or Medical Registry, takes BP‐lowering drugs, no drugs indicated for HT); (d) *Untreated HT/ White Coat Syndrome* (hypertensive resting BP at medical examination, diagnosis of HT and treatment according to examination or Medical Registry, no BP‐lowering drugs, no drugs indicated for HT); and (e) *Previous antihypertension treatment* (denies current but reports previous diagnosis of HT and treatment according to examination or Medical Registry, no present BP‐lowering drugs, no drugs indicated for HT; all cases had a hypertensive resting BP at doctor's examination). Sixteen cases were excluded due to the use of BP‐lowering drugs for an indication other than HT and no HT diagnosis; of these cases, seven participants were hypertensive, and nine were normotensive).

### 2.4 Magnetic resonance image acquisition and processing

All patients were examined with a three Tesla MRI (General Electric, discovery MR 750w). MR examinations included the following: axial T2‐weighted fluid‐attenuated inversion recovery (T2 FLAIR), axial diffusion‐weighted images (DWI), and axial susceptibility‐weighted angiography (SWAN). SWAN sequences were acquired at two settings: 3‐mm‐thick SWAN images and 5‐mm‐thick phase images in order to differentiate blood from calcifications. Furthermore, sagittal T1‐weighted 0.9‐mm isotropic 3D fast spoiled gradient echo (3D‐FSPGR) images were performed and reconstructed in the axial and coronal planes. MR images were assessed by an experienced neuroradiologist, and the MRI examinations were evaluated with regard to occurrence of the following: (a) white matter changes, classified according to Fazekas^16^ grading, and white matter changes in the brain stem, (b) lacunar infarcts (less than 1 cm in diameter), (c) CMB defined as small (2‐5 mm) hypointense lesions on MRI,^17^ (d) medial temporal atrophy (MTA), graded according to Scheltens’^18^ scale, and (e) specific types of atrophy, including frontal cortical, temporal cortical, frontotemporal (FTA), cerebellar, and midbrain atrophy. MRI diagnoses of CMB have proven to be reproducible with good to very good intra‐ and interrater agreement (intrarater kappa = 0.85 [95% confidence interval (CI) 0.77‐0.93]; interrater kappa = 0.68 [95% CI: 0.58‐0.78]) in previous studies.^19^ In our study, we examined the subjects with the SWAN sequence (corresponding to the DWI sequence) using thin slices, see above, at three Tesla magnetic field strength. Previous studies have shown that higher field strength, thin slices, and alternatives to conventional GRE MRI substantially improved CMB contrast and detection rate.^19^, ^20^

### 2.5 Statistical analysis

Demographic variables were selected a priori from the baseline examinations and questionnaires. Age cohorts were determined based on sample sizes. Possible correlations of background characteristics with CMB were initially investigated using Pearson chi‐square or Fisher's exact tests when appropriate and subsequently using univariate logistic regression analysis with “CMB yes/no” set as the dependent variable. The significance level was set to 0.05 and for the covariates, that is, sociodemographics, lifestyle, medical diagnosis, and medications, where the *P* < 0.05, a multivariate analysis adjusted for age and sex was performed. BP variables were investigated where CMB were stratified by localization as nonlobar and lobar. We examined the relationship between the number of CMB found and different aspects of HT using a linear regression model. The number of CMB was restricted to 0‐10 for graphical reasons, and therefore, cases with >=10 microbleeds were set as 10 microbleeds. IBM SPSS Statistics v.24 was used for analyses.

Informed consent was obtained from each patient. Ethical approval was obtained from the Ethical Committee at Lund University, 2015/859.

## 3 RESULTS

### 3.1 Baseline characteristics

Out of 344 subjects examined with MRI, 91 subjects had CMB (26.5%), of whom 61% had one, 31% had 2‐10, and 3% had 10‐99 CMB. Notably, 4% had more than 100 CMB, Table 2. The distribution of CMB was strictly lobar in 59% of subjects and nonlobar (combined, deep, cerebellar, pontine) in 37%. The prevalence of CMB increased from 18% among subjects in their 70s to 30% among those over 80 years old (odds ratio [OR] = 1.07; CI: 1.01‐1.14) (Table 1). The presence of CMB was associated with male sex (OR = 1.96; CI: 1.21‐3.18). After adjustment for sex and HT, age did not remain an independent confounder. Impaired cognitive function, defined by MMSE scores under 25, was associated with increased risk for CMB (OR = 4.0; CI: 1.66‐9.63). A mild‐moderate cognitive impairment (MMSE scores of 25‐27) was also associated with CMB (OR = 1.90; 1.10‐3.27). Angina pectoris was also associated with CMB after adjustment for age and sex (OR = 2.6; CI: 1.333‐5.11). Medication with anticoagulants was associated with CMB (OR = 1.67; CI: 1.01‐2.76) but was nonsignificant after adjustment for age and sex. No associations were noted between CMB and lifestyle factors or medical history of diabetes, atrial fibrillation, myocardial infarction, stroke, and depression.

**Table 2:** Prevalence of MRI findings (n, %) in the different age‐groups and the total cohort. Odds ratios (ORs) for coexisting CMB are from the multivariate logistic regression (adjusted for age and sex)

	Age			Total cohort	Multivariate		
	70‐74 y	75‐79 y	≥80 y				
	n = 128	n = 127	n = 89	n = 344	OR	95% CI	*P*
White matter hyperintensities (Fazekas score)				290 (84.3%)	2.06	0.95‐4.48	0.067
None	24 (18.8%)	18 (14.2%)	12 (13.5%)	54 (15.7%)	1	‐	‐
Mild/sporadic	70 (54.7%)	68 (53.5%)	41 (46.1%)	179 (52%)	1.71	0.76‐3.81	0.194
Moderate beginning confluent	23 (18.0%)	30 (23.6%)	20 (22.5%)	73 (21.2%)	1.52	0.60‐3.84	0.376
Severe confluent	11 (8.6%)	11 (8.7%)	16 (18.0%)	38 (11%)	7.02	2.62‐18.84	0.000
MTA‐ medial temporal lobe atrophy score				262 (76.2%)	1.26	0.69‐2.31	0.444
None	33 (25.8%)	27 (21.3%)	21 (23.6%)	81 (23.5%)	1	‐	‐
MTA 1	83 (64.8%)	74 (58.3%)	38 (42.7%)	195 (56.7%)	1.16	0.62‐2.17	0.650
MTA 2‐3	11 (8.7%)	26 (20.5%)	30 (33.7%)	67 (19.5%)	1.58	0.75‐3.36	0.231
MTA 4	0	0	0	0	‐	‐	‐
MTA score pathological for age (<75:>1, >75:>2)	11 (8.6%)	4 (3.1%)	8 (9%)	26 (7.6%)	1.94	0.84‐4.49	0.121
GCA ‐global cortical atrophy, 13 regions (Pasquier)				48 (14%)	0.72	0.34‐1.49	0.370
None	113 (88.3%)	112 (88.2%)	71 (79.8%)	296 (86%)	1	‐	‐
Mild GCA	15 (11.7%)	12 (9.4%)	13 (14.6%)	40 (11.6%)	0.68	0.30‐1.52	0.343
Moderate GCA	0	3 (2.4%)	5 (5.6%)	8 (2.3%)	0.90	0.20‐4.07	0.894
Severe GCA	0	0	0	0	‐	‐	‐
Specific atrophy	30 (23.4%)	40 (31.5)	30 (33.7%)	100 (29.1%)	0.53	0.30‐0.94	0.030
White matter changes, pontine	17 (13.3%)	21 (16.5%)	17 (19.1%)	55 (16%)	1.84	0.98‐3.45	0.057
Cerebral infarctions	18 (14.1%)	15 (11.8%)	14 (15.7%)	47 (13.6%)	0.89	0.44‐1.84	0.763
Lacunar infarctions	12 (9.5%)	10 (7.8%)	11 (12.2%)	33 (9.5%)	1.03	0.46‐2.32	0.936
Cerebral microbleeds (CMB)	24 (18.8%)	40 (31.5%)	27 (30.3%)	91 (26.3%)	‐	‐	‐
Quantity							
1 microbleed	12 (9.4%)	28 (22%)	16 (18%)	56 (61.5%)	‐	‐	‐
2‐10 microbleeds	10 (41.7%)	10 (25%)	8 (9%)	28 (30.8%)	‐	‐	‐
10‐99 microbleeds	1 (0.8%)	0	2 (2.2%)	3 (3.3%)	‐	‐	‐
≥100 microbleeds	1 (0.8%)	2 (1.6%)	1 (1.1%)	4 (4.4%)	‐	‐	‐
Location							
Lobar	15 (11.7%)	22 (17.3%)	17 (19.1%)	54 (59.3%)	‐	‐	‐
Deep	2 (1.6)	6 (4.7%)	2 (2.2%)	10 (11%)	‐	‐	‐
Cerebellum	2 (1.6%)	5 (3.9%)	4 (4.5%)	11 (12.1%)	‐	‐	‐
Combined lobar/deep	2 (1.6%)	2 (1.6%)	3 (3.4%)	7 (7.7%)	‐	‐	‐
Pons/other	1 (0.8%)	3 (1.6)	1 (1.1%)	5 (5.5%)	‐	‐	‐
Location information missing				4 (4.4%)			

### 3.2 MRI findings

All grades of white matter hyperintensities (WMH) were observed in 84% of the study group, while the proportion of severe grades increased from 8.6% among subjects in their 70s to 18% among those over 80 years old (Table 2). The proportion of MTA grades 2‐3 also increased with age, from 8.7% in the youngest subgroup to 33.7% in the oldest subgroup. A similar trend, but lower prevalence, was observed for global cortical atrophy. Lacunar infarcts and cerebral infarcts were observed in 9.5% and 13.6% of all cases, respectively, and were not associated with age. Severe WMH, defined as a Fazekas score of 3, were associated with CMB (OR = 7.02; CI: 2.62‐18.84). Global cortical atrophy, MTA, or cerebral or lacunar infarcts were associated with CMB.

### 3.3 Association between CMB and blood pressure measurements

In the model adjusted for age and sex, CMB were associated with systolic and diastolic HT and peripheral pulse pressure (PPP) but not with ABI or orthostatic hypotension (Table 3). After stratification by nonlobar and lobar CMB, the associations between HT, systolic HT, diastolic HT, and increased PPP remained significant with the nonlobar CMB, but not with the lobar CMB (Table 4). A 1 mm Hg increase in PPP corresponded to an OR = 1.03 (CI: 1.01‐1.05) of the nonlobar CMB, while moderately increased PPP over 80 mm Hg showed an OR = 3.17 (CI: 1.25‐8.02). There was no association between the number of HT treatment years and the presence of CMB. In a linear regression model adjusted for age, no associations were found between the scale variables of systolic and diastolic BP, PPP, number of HT treatment years, and number of CMB (data not shown).

**Table 3:** Peripheral blood pressure measurement characteristics. Odds ratios (ORs) for coexisting CMB are from the multivariate logistic regression analysis (adjusted for age and sex)

	n	Multivariate		
		OR	95% CI	*P*
Systolic blood pressure (SBP) mean (median), range	144 (142), 98‐210	1.01	1.00‐1.03	0.062
Systolic hypertension	193 (56.1%)	1.69	1.01‐2.83	0.048
Missing	4			
Diastolic blood pressure (DBP) mean (median), range	78 (79), 45‐100	1.01	0.98‐1.04	0.492
Diastolic hypertension	37 (10.8%)	2.26	1.11‐4.61	0.025
Missing	10			
Hypertension (SBP or DBP)	200 (58.1%)	1.93	1.13‐3.28	0.016
Peripheral pulse pressure, mm Hg, left arm mean (median), range	65 (62), 25‐128	1.01	1.00‐1.03	0.083
0‐60	157 (45.6%)	1	‐	‐
61‐80	108 (31.4%)	2.12	1.19‐3.78	0.011
>81	69 (20.1%)	1.84	0.95‐3.56	0.072
Ankle‐brachial index (ABI), left side mean (median), range	1.13 (1.14), 0.51‐1.74	0.62	0.12‐3.30	0.573
ABI < 0.9	23 (6.6%)	1.28	0.49‐3.34	0.614
ABI 0.9‐1.29	270 (78.0%)	1	‐	‐
ABI ≥ 1.3	35 (10.1%)	0.57	0.24‐1.37	0.209
Orthostatic intolerance with symptoms at tilting the past year	106 (30.8%)	1.22	0.72‐2.05	0.465
Orthostatic hypotension at tilting during medical examination	36 (10.5%)	0.90	0.40‐2.01	0.800
BP phenotypes				
Healthy controls^a^	72 (20.9%)	1	‐	‐
Controlled hypertension^b^	63 (18.3%)	1.49	0.63‐3.54	0.368
Uncontrolled hypertension^c^	92 (26.7%)	2.30	1.05‐5.05	0.037
Untreated hypertension/ WCS^d^	90 (26.2%)	2.02	0.91‐4.51	0.085
Previous antihypertensive treatment^e^	10 (2.9%)	2.23	0.48‐10.36	0.307
Missing/excluded	17 (4.9%)			
Duration of antihypertensive treatment mean (median), range	14.2 (12), 2‐54			
No treatment	185 (53.8%)	1.10	0.56‐2.18	0.780
1‐10 y	66 (19.2%)	1	‐	‐
11‐20 y	52 (15.1%)	1.64	0.72‐3.72	0.235
>20 y	28 (8.1%)	1.99	0.76‐5.23	0.162

Abbreviation: HT, hypertension, WCS, white coat syndrome

a No present or previous antihypertensive treatment, no BP‐lowering drugs, no drugs indicated for HT, not hypertensive at medical examination.

b Reports antihypertensive treatment, takes BP‐lowering drugs with an indication for HT, not hypertensive at medical examination.

c Reports antihypertensive treatment, takes BP‐lowering drugs with an indication for HT, hypertensive at medical examination.

d No present or previous antihypertensive treatment, no BP‐lowering drugs, no drugs indicated for HT, hypertensive at medical examination.

e No present antihypertensive treatment, reports previous treatment for HT, no BP‐lowering drugs, no drugs indicated for HT. All cases are hypertensive at medical examination.

**Table 4:** Blood pressure measurements and relation to lobar and nonlobar CMB. Odds ratios (ORs) for coexisting CMB are from the multivariate logistic regression analysis (adjusted for age and sex). In the nonlobar subcohort, all cases with strictly lobar CMB have been excluded. In the lobar subcohort, all cases with deep, mixed or cerebellar CMB have been excluded

	n	Multivariate (n = 290) Nonlobar subcohort			n cases	Multivariate (n = 311) Lobar subcohort			Lobar CMB n = 54	Nonlobar CMB n = 33
		OR	95% CI	*P*		OR	95% CI	*P*		
Systolic blood pressure (SBP) mean (median), range	144 (142), 98‐210	1.03	1.01‐1.05	0.007	143 (142) 98‐210	1.01	0.99‐1.03	0.258	143 (144) 100‐172	149.5 (147) 122‐186
Systolic hypertension	161 (55.5%)	2.17	1.00‐4.69	0.049	171 (55%)	1.65	0.89‐3.04	0.110	32 (59.3%)	22 (66.7%)
Missing	3 (1%)	‐	‐	‐	4 (1.3%)	‐	‐	‐	1 (1.9%)	0
Diastolic blood pressure (DBP) mean (median), range	78 (79), 45‐98	1.02	0.98‐1.06	0.392	78 (79) 45‐100	1.01	0.98‐1.05	0.452	78 (78), 56‐100	78 (79) 50‐98
Diastolic hypertension	28 (9.7%)	2.69	1.04‐6.96	0.042	32 (10.3%)	2.51	1.11‐5.63	0.026	9 (16.7%)	5 (15.2%)
Missing	9 (3.1%)	‐	‐	‐	9 (2.9%)	‐	‐	‐	1 (1.9%)	1 (3%)
Hypertension (SBP or DBP)	167 (57.6%)	3.11	1.33‐7.28	0.009	176 (56.6%)	1.67	0.90‐3.09	0.105	33 (61.1%)	24 (72.7%)
Peripheral pulse pressure, mm Hg left arm, mean (median), range	65 (62), 25‐128	1.03	1.01‐1.05	0.014	64 (61) 25‐128	1.01	0.99‐1.03	0.338	64 (64), 40‐91	70 (69), 43‐103
0‐60	135 (46.6%)	1	‐	‐	147 (47.3%)	1	‐	‐	22 (40.7%)	10 (30.3%)
61‐80	87 (30%)	2.72	1.12‐6.61	0.027	97 (31.2%)	2.13	1.09‐4.14	0.027	21 (38.9%)	11 (33.3%)
>81	59 (20.3%)	3.17	1.25‐8.02	0.015	58 (18.6%)	1.43	0.63‐3.27	0.392	10 (18.5%)	11 (33.3%)
Ankle‐brachial index (ABI), left side mean (median), range	1.12 (1.14) 0.51‐1.74	0.13	0.01‐1.45	0.098					1.16 (1.17), 0.59‐1.56	1.09 (1.09) 0.85‐1.33
ABI < 0.9	20 (6.9%)	1.83	0.56‐6.01	0.322	19 (6.1%)	0.84	0.23‐3.09	0.792	3 (5.6%)	4 (12.1%)
ABI 0.9‐1.29	227 (78.3%)	1	‐	‐	245 (78.8%)	1	‐	‐	43 (79.6%)	25 (75.8%)
ABI ≥ 1.3	29 (10%)	0.36	0.08‐1.67	0.191	33 (10.6%)	0.65	0.24‐1.71	0.379	6 (11.1%)	2 (6.1%)
Orthostatic intolerance with symptoms at tilting the past year	88 (30.3%)	1.24	0.59‐2.58	0.574	94 (30.2)	1.20	0.64‐2.25	0.561	18 (33.3%)	12 (36.4%)
Orthostatic hypotension at tilting during medical examination	30 (10.3%)	0.84	0.27‐2.63	0.758	32 (10.3%)	0.88	0.34‐2.32	0.803	6 (11.1%)	4 (12.1%)
BP phenotypes										
Healthy controls^a^	61 (21%)	1	‐	‐	71 (22.8%)	1	‐	‐	11 (20.4%)	1 (3%)
Controlled hypertension^b^	55 (19%)	9.18	1.09‐77.14	0.041	55 (17.7%)	0.84	0.31‐2.32	0.738	8 (14.8%)	8 (24.2%)
Uncontrolled hypertension^c^	77 (26.6%)	14.40	1.82‐114.10	0.012	79 (25.4%)	1.54	0.65‐3.64	0.326	15 (27.8%)	13 (39.4%)
Untreated hypertension/ WCS^d^	75 (25.9%)	9.08	1.09‐75.52	0.041	82 (26.4%)	1.53	0.64‐3.66	0.336	15 (27.8%)	8 (24.2%)
Previous antihypertensive treatment^e^	8 (2.8%)	10.63	0.58‐195.82	0.112	9 (2.9%)	1.65	0.29‐9.26	0.576	2 (3.7%)	1 (3%)
Missing/excluded	14 (4.8%)	‐	‐	‐	15 (4.8%)	‐	‐	‐	3 (5.6%)	2 (6.1%)
Duration of antihypertensive treatment, mean (median), range	14 (12) 2‐54	1.03	0.99‐1.08	0.196	13.7 (12) 2‐54	1.01	0.97‐1.06	0.602	14.9 (13), 2‐45	17 (14), 6‐40
No treatment	155 (53.4%)	0.59	0.23‐1.49	0.587	173 (55.6%)	1.55	0.65‐3.68	0.324	30 (55.6%)	12 (36.4%)
1‐10 y	59 (20.3%)	1	‐	‐	58 (18.6%)	1	‐	‐	7 (13%)	8 (24.2%)
11‐20 y	42 (14.5%)	1.36	0.47‐3.92	0.570	46 (14.8%)	2.15	0.78‐5.93	0.137	10 (18.5%)	6 (18.2%)
>20 y	24 (8.3%)	2.20	0.70‐6.90	0.178	21 (6.8%)	1.52	0.40‐5.80	0.544	4 (7.4%)	7 (21.2%)

Abbreviation: HT, hypertension; WCS = white coat syndrome.

a No present or previous antihypertensive treatment, no BP‐lowering drugs, no drugs indicated for HT, not hypertensive at medical examination.

b Reports antihypertensive treatment, takes BP‐lowering drugs with an indication for HT, not hypertensive at medical examination.

c Reports antihypertensive treatment, takes BP‐lowering drugs with an indication for HT, hypertensive at medical examination.

d No present or previous antihypertensive treatment, no BP‐lowering drugs, no drugs indicated for HT, hypertensive at medical examination.

e No present antihypertensive treatment, reports previous treatment for HT, no BP‐lowering drugs, no drugs indicated for HT. All cases are hypertensive at medical examination.

### 3.4 BP phenotypes

The uncontrolled HT group (n = 92) had an increased risk of CMB compared to the normotensives (n = 72) (OR = 2.30; CI: 1.05‐5.05; *P* = 0.037; in the multivariate model). When adjusted for age and sex, a significantly higher OR of nonlobar CMB was observed in subjects with controlled HT, uncontrolled HT, and untreated HT than in the healthy controls (Table 4). Intake of the drugs anticoagulants (*P* = 0.047) and Ca‐channel blockers (*P* = 0.023) was associated to CMB, OR = 1.67, respectively OR = 1.98, in univariate analyses, see Table S1. Medial temporal lobe atrophy score was associated to CMB, OR = 2.08, (*P* = 0.046). Age‐corrected MTA score showed a similar association, OR = 2.18, (*P* = 0.062), see Table S2. The pattern between blood pressure measurements and CMB was similar in univariate and multivariate analyses, Table S3.

## 4 DISCUSSION

In our study, we investigated the relationships between different HT treatment conditions and the risk of CMB. The prevalence of CMB strongly increased with age, and in most cases, there was a single instance with a lobar localization. The main effect of HT on the prevalence of CMB was observed in nonlobar CMB, especially among subjects with uncontrolled HT.^5^, ^8^ CMB were associated with WMH and cognitive decline but not with other manifestations of cerebrovascular disease or atrophies.

The prevalence of CMB in this study (18.8% in the younger elderly of the study, aged 70‐74 years) is somewhat higher than in the Framingham Heart Study (8.8%, mean age: 66.5 years),^7^ the AGES‐Reykjavik study (11.1%‐15.3%, mean age: 76 years),^5^ the Swedish PIVUS (14%, mean age: 75 years),^21^ and a recent Japanese population study of elderly people (23%).^22^ Some of the differences could be attributed to a younger study population, selection bias with healthier participants, and MRI field strength used. For example, in the PIVUS study, only 7% of study subjects had an MMSE score below 28, compared to 35% in the present study. Comparable results to our data were reported by the Rotterdam Study, where the prevalence of CMB was 18% among those 60‐69 years old, and 38% in those over 80 years old,^3^ as well as in the ARIC study (24% with CMB, mean age: 76 years), which had a similar proportion of their sample with impaired cognition (36%).^23^

Subjects with uncontrolled HT had a higher risk of CMB than normotensives. These data suggest that well‐regulated antihypertensive treatment is protective against CMB. However, in the nonlobar subcohort, having controlled HT was associated with an increased risk of CMB. This suggests that the pathological mechanisms underlying HT, rather than the BP itself, might contribute to the development of CMB. Another possible explanation is that the pathophysiological mechanisms differ among HT populations, rendering hypertensive subjects with hard to normalize BP levels with a higher risk of cerebral microangiopathy. Severe confluent WMH were related to impaired cognition, and the observed association between CMB and the MMSE is well in line with the covariance between CMB and WMH.

It is not fully understood what aspect of HT and its mechanisms induce CMB. One theory is that increased arterial stiffness, both an effect of and a contributor to HT, is the main pathological mechanism behind CSVD and CMB. It is believed that the decrease in arterial autoregulation following arterial stiffness makes them less compliant to the pulse wave, which propagates the pulse wave further down the arterial tree. This might explain the main effect of HT, controlled as well as uncontrolled, on nonlobar but not lobar CMB. It seems that organs with penetrating arterioles, such as brain, kidney, and retina, are more sensitive to these changes, likely because they are more exposed to the propagated pulse wave and the resulting mechanical damage of the organ. Different pathological mechanisms could contribute to the effect of HT in nonlobar CMB with a higher proportion of arterioles with smooth muscle and the subsequent development of atheroma and reduced vessel lumen compared to the capillary level of end arterioles in the lobar regions with effects on endothelium and pericytes. CSVD has also been associated with chronic kidney disease,^24^ and a decreased estimated glomerular filtration rate (eGFR) and arterial stiffness have been associated with cognitive impairment.

Many studies have investigated the effect of HT (defined as a heterogeneous group including increased resting BP, self‐reported diagnosis, and/or antihypertensive treatment), but few studies have tried to investigate the different aspects of HT in detail. In our study, we found that an increase in PPP leads to an increased risk of nonlobar CMB. ABI can be viewed as a proxy for both atherosclerosis (<0.9) and extensive peripheral arterial stiffness (>1.3). However, in this study, no associations were noted in any direction between pathological ABI and CMB.

The association between age and CMB disappeared after adjusting for sex and HT. This is inconsistent with most previous studies. However, previous data were based on younger cohorts. It is possible that the increased effect of age on CMB is saturated by introducing a survival bias, as an increasing number of individuals with less favorable genetic or lifestyle phenotypes have died or failed to come to examinations due to illness. Another contributing factor to that inconsistency is that no other studies included HT in the multivariate analyses, as the impact of this risk factor increases with age.

It is noteworthy that 22% of the participants above 80 years old had a history of atrial fibrillation and 44% of them used anticoagulants. However, CMB have been associated with both IS and ICH, especially in Asian populations and in subjects with lobar CMB associated with CAA.^25^ In patients with nonvalvular atrial fibrillation, the presence of five or more CMB was an independent predictor of all‐cause mortality and IS mortality, while patients with lobar CMB had increased risk for ICH (hazard ratio: 5.91).^26^ A study comparing patients with ICH vs. IS while taking nonvitamin K antagonists showed that the ICH group had a higher proportion of CMB (79% vs 37%) and a higher number of CMB (median: 5 vs 0).^27^ Further studies are needed to interpret the risk association between CMB and decisions of anticoagulant treatment.

A strength of the present study is its design—a large, general population‐based study, with randomized inclusion of participants and a high proportion of subjects over 80 years of age. Home visits were offered at re‐examinations to reduce selection bias. Although the selection of participants for the MRI was randomized from the GÅS study, the inclusion criteria were restricted to subjects able to perform the MRI and clinical investigation, thereby excluding the frail and demented. However, since the study comprises relatively healthy subjects, the results are to a large extent transferrable to the normal elderly urban population. A notable study limitation was that it was conducted in a northern European country on a population with little ethnic diversity, possibly reducing applicability to other ethnic groups.

## 5 SUMMARY

CMB are an independent marker of CSVD, and the presence of nonlobar CMB is explained mainly by uncontrolled HT. CMB are frequent in the general older population, about one‐third of those over 75 years of age, and are associated with WMH and cognitive impairment. These results indicate the need to implement recommended guidelines for HT treatment in the elderly population, although further prospective studies are needed to address causality, especially the effects of central BP, arterial stiffness, and pulse wave propagation on occurrence of CMB.

## CONFLICT OF INTEREST

No conflicts of interest to disclose.

## AUTHOR CONTRIBUTION

All authors contributed to the study with substantial contributions to the conception or design of the work, or the acquisition, analysis, or interpretation of data for the work; drafting the work or revising it critically for important intellectual content; final approval of the version to be published; and agreement to be accountable for all aspects of the work in ensuring that questions related to the accuracy or integrity of any part of the work are appropriately investigated and resolved.
