# Supplementary material for: Association between cerebral microbleeds and hypertension in the Swedish general population “Good Aging in Skåne” study
Source: J Clin Hypertens (Greenwich). 2019 Jul 5;21(8):1099–107. doi: 10.1111/jch.13606 (PMC6771849; doi:10.1111/jch.13606)
Supplement: Supplementary file 1 [file JCH-21-1099-s001.docx]

Supplemental tables

**Table I**

Baseline characteristics of study participants according to age groups and the total cohort. The prevalence of CMB and Odds ratios (OR) for coexisting CMB are given from univariate and multivariate logistic regression analysis.

|  | Age groups n (%) | | | Total cohort n=344: | | | | | | | |
| --- | --- | --- | --- | --- | --- | --- | --- | --- | --- | --- | --- |
|  | 70 – 74 yrs | 75 – 79 yrs | > 80 yrs | Cases | Prevalence  of CMB | Univariate | | | Multivariate^A^ | | |
|  | 128 | 127 | 89 | n (%) | n (%) | OR | 95 % CI | p | OR | 95 % CI | p |
| Age mean (median), range |  |  |  | 77 (76),70 -87 | 91 (26.5%) | 1,07 | 1,01-1,14 | 0,026 | 1,06 | 0,99 – 1,13 | 0,099 |
| 70-74 yrs |  |  |  | 128 (37,2%) | 24 (18.8%) | 1 | - | - | 1 | - | - |
| 75-79 yrs |  |  |  | 127 (36,9%) | 40 (31.5%) | 1,89 | 1,00-3,56 | 0,049 | 2,03^B^ | 0,85 – 4,83 | 0,109 |
| 80-87 yrs |  |  |  | 89 (25,9%) | 27 (30.3%) | 1,99 | 1,12-3,56 | 0,020 | 1,44^B^ | 0,53 – 3,88 | 0,477 |
| female | 76 (59,4%) | 70 (55,1%) | 51 (57,3%) | 197 (57,3%) | 41 (20.8%) | 1 | - | - | 1 | - | - |
| male | 52 (40,6%) | 57 (44,9%) | 38 (42,7%) | 147 (42,7%) | 50(34%) | 1,96 | 1,21-3,18 | 0,006 | 2,27^C^ | 1,37– 3,77 | 0,002 |
| Living alone | 45 (35,2%) | 77 (60,6%) | 39 (43,8%) | 134 (39%) | 37 (27.6%) | 1 | - | - |  |  |  |
| Cohabitant | 83 (64,8%) | 50 (39,4%) | 50 (56,2%) | 210 (61%) | 54 (25.7%) | 0,91 | 0,56-1,48 | 0,697 |  |  |  |
| Elementary school or less | 36 (28,1%) | 44 (34,6%) | 34 (38,2%) | 114 (33,1%) | 32 (28,1%) | 1 | - | - |  |  |  |
| Secondary school | 56 (43,8%) | 27 (21,3%) | 25 (28,1%) | 108 (31,4%) | 29 (26.9%) | 0,94 | 0,52 - 1,70 | 0,839 |  |  |  |
| University > 1 year | 36 (28,1%) | 24 (18,9%) | 28 (31,5%) | 88 (25,6%) | 18 (20.5%) | 0,66 | 0,34 - 1,27 | 0,215 |  |  |  |
| Independent ADL | 117 (91,4%) | 120(94,5%) | 79 (88,8%) | 316 (91,9%) | 81 (25.6%) | 1 | - | - |  |  |  |
| Dependent IADL or PADL | 10 (7,8%) | 7 (5,5%) | 8 (9,0%) | 25 (7,3%) | 9 (36%) | 1,63 | 0,694 - 3,84 | 0,261 |  |  |  |
| Never smoked | 43 (33,6%) | 48 (37,8%) | 37 (41,6%) | 128 (37,2) | 35 (27.3%) | 1 | - | - |  |  |  |
| former smoker | 62 (48,4%) | 64 (50,4%) | 47 (52,8%) | 173 (50,3%) | 48 (27.7%) | 1,02 | 0,6 - 1,70 | 0,939 |  |  |  |
| Smoker | 22 (17,2%) | 14 (11,0%) | 5 (5,6%) | 41 (11,9%) | 8 (19.5%) | 0,64 | 0,27 - 1,53 | 0,319 |  |  |  |
| No alcohol since > 1 year | 11 (8,6%) | 12 (9,4%) | 12 (13,5%) | 35 (10,2%) | 8 (22.9%) | 1 | - | - |  |  |  |
| Alcohol this year but not this month | 10 (7,8%) | 15 (11,8%) | 12 (13,5%) | 37 (10,8%) | 11 (29,7%) | 1,43 | 0,50 - 4,11 | 0,509 |  |  |  |
| Alcohol this month | 106 (82,8%) | 99 (78,0%) | 65 (73,0%) | 270 (78,5%) | 72 (26.7%) | 1,23 | 0,53 - 2,83 | 0,630 |  |  |  |
| Sedentary /mostly sitting /occasional slow walks | 9 (7,0%) | 12 (9,4%) | 6 (6,7%) | 27 (7,8%) | 7 (25.9%) | 1 | - | - |  |  |  |
| Light exercise 2-4hrs/week | 60 (46,9%) | 77 (60,6%) | 50 (56,2%) | 187 (54,4%) | 52 (27.8%) | 1,10 | 0,440 - 2,76 | 0,838 |  |  |  |
| Moderate exercise, e.g. jogging, 1-2 hrs/week | 47 (36,7%) | 31 (24,4%) | 25 (28,1%) | 103 (29,9%) | 21 (20.4%) | 0,73 | 0,27 - 1,96 | 0,534 |  |  |  |
| Hard exercise > 1/week or moderate exercise > 2 hrs | 10 (7,8%) | 6 (4,7%) | 6 (6,7%) | 22 (6,4%) | 9 (40.9%) | 1,98 | 0,59 - 6,63 | 0,269 |  |  |  |
| < 8 hours sleep on average | 58 (45,3%) | 60 (47,2%) | 50 (56,2%) | 168 (48,8%) | 46 (27.4%) | 1,29 | 0,75 - 2,22 | 0,352 |  |  |  |
| 8 hours sleep on average | 49 (38,3%) | 46 (36,2%) | 29 (32,6%) | 124 (36,0%) | 28 (22.6%) | 1 | - | - |  |  |  |
| > 8 hours sleep on average | 19 (14,8%) | 20 (15,7%) | 8 (9,0%) | 47 (13,7%) | 15 (31.9%) | 1,61 | 0,76 - 3,38 | 0,211 |  |  |  |
| Answered ”yes” on: |  |  |  |  |  |  |  |  |  |  |  |
| Hard time falling asleep | 28 (21,9%) | 33 (26%) | 24 (27,0%) | 85 (24,7%) | 19 (21.1%) | 0,75 | 0,42 - 1,34 | 0,330 |  |  |  |
| Use insomnia medication | 13 (10,2%) | 17 (13,4%) | 16 (18,0%) | 46 (13,4%) | 11 (23.9%) | 0,87 | 0,42 - 1,79 | 0,698 |  |  |  |
| Wake up early | 62 (48,4%) | 70 (55,1%) | 60 (67,4%) | 192 (55,8%) | 53 (27.6%) | 1,18 | 0,72 - 1,92 | 0,519 |  |  |  |
| Sleep apnea or needs to sleep > 2 hrs daytime | 14 (10,9%) | 13 (10,2%) | 5 (5,6%) | 32 (9,3%) | 8 (25%) | 0,93 | 0,40 - 2,15 | 0,866 |  |  |  |
| **Cognitive performance:** |  |  |  |  |  |  |  |  |  |  |  |
| I have not noticed any loss of memory in recent years | 39 (30,5%) | 41 (32,2%) | 22 (24,7%) | 102 (29,7%) | 21 (20.6%) | 1 | - | - |  |  |  |
| I have noticed loss of memory to some degree | 81 (63,3%) | 78 (61,4%) | 55 (61,8%) | 214 (62,2%) | 64 (29.9%) | 1,65 | 0,94 - 2,89 | 0,082 |  |  |  |
| I have noticed a significant loss of memory | 7 (5,5%) | 8 (6,3%) | 10 (11,2%) | 25 (7,3%) | 5 (20.0%) | 0,96 | 0,32 - 2,87 | 0,964 |  |  |  |
| 5-item immediate and delayed answers |  |  |  |  |  |  |  |  |  |  |  |
| Correct | 36 (28,1%) | 38 (29,9%) | 30 (33,7%) | 104 (30,2%) | 21 (20.2%) | 1 | - | - |  |  |  |
| 1 false answer on either | 80 (62,5%) | 70 (55,1%) | 42 (47,2% | 192 (55,8%) | 55 (28.6%) | 1,59 | 0,87 - 2,81 | 0,114 |  |  |  |
| > 2 false answer on either | 10 (7,8%) | 19 (15%) | 15 (16,9%) | 44 ( 12,8%) | 14 (31.8%) | 1,84 | 0,83 - 4,08 | 0,131 |  |  |  |
| MMSE score |  |  |  |  |  |  |  |  |  |  |  |
| 28-30 | 90 (70,3%) | 81 (63,8%) | 53 (59,6%) | 224 (65,1%) | 48 (21.4%) | 1 | - | - | 1 | - | - |
| 25-27 | 26 (20,3%) | 40 (31,5%) | 22 (24,7%) | 88 (25,6%) | 30 (34.1%) | 1,90 | 1,10 - 3,27 | 0,021 | 1,87 | 1,07 - 3,25 | 0,028 |
| < 24 | 8 (6,3%) | 6 (4,7%) | 9 (10,1%) | 23 (6,7%) | 12 (52.2%) | 4,00 | 1,66 - 9,63 | 0,002 | 3,87 | 1,57 - 9,51 | 0,003 |
| **History of medical diagnosis** |  |  |  |  |  |  |  |  |  |  |  |
| depression | 25 (19,5%) | 17 (13,4%) | 13 (14,6%) | 55 (16%) | 13 (23,6%) | 0,84 | 0,43 - 1,65 | 0,613 |  |  |  |
| dementia | 3 (2,3%) | 2 (1,6%) | 1 (1,1%) | 6 (1,7%) | 1 (16,7%) | 0,55 | 0,06 - 4,78 | 0,589 |  |  |  |
| diabetes mellitus | 23 (18%) | 10 (7,9%) | 10 (11,2%) | 43 (12,5%) | 13 (30,2%) | 1,24 | 0,61 - 2,51 | 0,542 |  |  |  |
| angina pectoris | 14 (10,9%) | 13 (10,2%) | 16 (18%) | 43 (12,5%) | 20 (46,5%) | 2,83 | 1,47 - 5,46 | 0,002 | 2,61 | 1,33 - 5,11 | 0,005 |
| myocardial infarction | 4 (3,1%) | 12 (9,4%) | 18 (20,2%) | 34 (9,9%) | 11 (32,4%) | 1,38 | 0,64 - 2,96 | 0,408 |  |  |  |
| atrial fibrillation | 7 (5,5%) | 16 (12,6%) | 20 (22,5%) | 43 (12,5%) | 16 (16,7%) | 1,59 | 0,81 - 3,14 | 0,179 |  |  |  |
| stroke/hemorrhage/TIA | 13 (10,2%) | 10 (7,9%) | 10 (11,2%) | 33 (9,6%) | 12 (13,3%) | 1,69 | 0,79 - 3,58 | 0,175 |  |  |  |
| Chronic obstructive pulmonary disease | 24 (18,8%) | 18 (14,2%) | 10 (11,2%) | 52 (15%) | 9 (17,3%) | 0,54 | 0,25 - 1,15 | 0,111 |  |  |  |
| **Medication at**  **re-examination *** |  |  |  |  |  |  |  |  |  |  |  |
| B01 - anticoagulants | 25 (19,5%) | 40 (31,5%) | 39 (43,8%) | 104 (30,2%) | 35 (33.7%) | 1,67 | 1,01 - 2,76 | 0,047 | 1,39 | 0,82 - 2,35 | 0,224 |
| C02 - antihypertensives | 1 (0,8%) | 2 (1,6%) | 1 (1,1%) | 4 (1,2%) | 1 (25%) | 0,93 | 0,10 - 9,02 | 0,947 |  |  |  |
| C03 - diuretics | 15 (11,7%), | 13 (10,2%) | 15 (16,9%) | 43 (12,5%) | 15 (34.9%) | 1,59 | 0,80 - 3,13 | 0,183 |  |  |  |
| C04 – periph. vasodilator | 0 | 0 | 0 | 0 | 0 | - | - | - |  |  |  |
| C05 - vasoprotectives | 0 | 0 | 0 | 0 | 0 | - | - | - |  |  |  |
| C07 - beta-blocking agents | 26 (20,3%), | 30 (23,6%), | 29 (32,6%), | 85 (24,7%) | 24 (28.2%) | 1,13 | 0,65 - 1,95 | 0,668 |  |  |  |
| C08 - Ca-channel blockers | 19 (14,8%) | 22 (17,3%) | 19 (21,3%) | 60 (17,4%) | 23 (38.3%) | 1,98 | 1,10 - 3,55 | 0,023 | 1,79 | 0,98 - 3,26 | 0,059 |
| C09 - renin-angiotensin system | 32 (25,0%) | 42 (33,1%) | 32 (36%) | 107 (31.1%) | 35 (32.7%) | 1,57 | 0,95 - 2,60 | 0,078 |  |  |  |
| C10 - lipid modifying | 45 (35,2%) | 42 (33,1%) | 38 (42,7%) | 125 (36,3%) | 39 (31.2%) | 1,46 | 0,89 - 2,38 | 0,133 |  |  |  |
| BP-lowering medication (C02, C03, C07, C08, C09) | 52 (40,6%) | 63 (49,6%) | 56 (62,9%) | 171 (49,7%) | 52 (30.4%) | 1,50 | 0,93 - 2,43 | 0,099 |  |  |  |
| medical indication hypertension | 46 (35,9%) | 54 (42,5%) | 46 (51,7%) | 146 (42,4%) |  | 1,47 | 0,91 - 2,38 | 0,116 |  |  |  |

^A^ if not noted otherwise; multivariate regression analysis with age and gender as model covariates.

^B^  Multivariate logistic regression model: gender and hypertension as model covariates.

^C^  Multivariate logistic regression model: age and hypertension as model covariates.

*Medication regardless of indication. Classification according to ATC (Anatomic Therapeutic Chemical classification system).

###### Table II

Prevalence of findings (%) on MRI in the different age groups and the total cohort. Odds ratios (OR) for coexisting CMB are given from univariate and multivariate logistic regression analysis.

|  | Age groups | | |  | | | | | | |
| --- | --- | --- | --- | --- | --- | --- | --- | --- | --- | --- |
|  | 70 – 74 yrs | 75 – 79 yrs | > 80 yrs | Total cohort | Univariate | | | Multivariate^A^ | | |
|  | n = 128 | n = 127 | n = 89 | n = 344 | OR | 95 % CI | p | OR | 95 % CI | p |
| **WMH White matter hyperintensities** Fazekas score |  |  |  | 290 (84,3%) | 1,97 | 0,92 - 4,22 | 0,080 | 2,06 | 0,95 - 4,48 | 0,067 |
| None | 24 (18,8%) | 18 (14,2%) | 12 (13,5%) | 54 (15,7%) | 1 | - | - | 1 | - | - |
| Mild/sporadic | 70 (54,7%) | 68 (53,5%) | 41 (46,1%) | 179 (52%) | 1,63 | 0,74 - 3,60 | 0,227 | 1,71 | 0,76 - 3,81 | 0,194 |
| Moderate beginning confluent | 23 (18,0%) | 30 (23,6%) | 20 (22,5%) | 73 (21,2%) | 1,40 | 0,57 - 3,47 | 0,463 | 1,52 | 0,60 - 3,84 | 0,376 |
| Severe confluent | 11 (8,6%) | 11 (8,7%) | 16 (18,0%) | 38 (11%) | 6,88 | 2,63 - 18,01 | 0,000 | 7,02 | 2,62 - 18,84 | 0,000 |
| **MTA** – **Medial Temporal Lobe Atrophy** score |  |  |  | 262 (76,2%) | 1,33 | 0,74 - 2,39 | 0,348 | 1,26 | 0,69 - 2,31 | 0,444 |
| none | 33 (25,8%) | 27 (21,3%) | 21 (23,6%) | 81 (23,5%) | 1 | - | - | 1 | - | - |
| MTA 1 | 83 (64,8%) | 74 (58,3%) | 38 (42,7%) | 195 (56,7%) | 1,11 | 0,60 - 2,06 | 0,738 | 1,16 | 0,62 - 2,17 | 0,650 |
| MTA 2-3 | 11 (8,7%) | 26 (20,5%) | 30 (33,7%) | 67 (19,5%) | 2,08 | 1,01 - 4,28 | 0,046 | 1,58 | 0,75 - 3,36 | 0,231 |
| MTA 4 | 0 | 0 | 0 | 0 | - | - | - | - | - | - |
| MTA score pathological for age (<75 yrs:>1, >75 yrs :>2) | 11 (8,6%) | 4 (3,1%) | 8 (9%) | 26 (7,6%) | 2,18 | 0,96 - 4,95 | 0,062 | 1,94 | 0,84 - 4,49 | 0,121 |
| **GCA** – **Global Cortical Atrophy** 13 regions (Pasquier) |  |  |  | 48 (14%) | 0,92 | 0,45 - 1,85 | 0,806 | 0,72 | 0,34 - 1,49 | 0,370 |
| None | 113 (88,3%) | 112(88,2%) | 71 (79,8%) | 296 (86%) | 1 | - | - | 1 | - | - |
| Mild GCA | 15 (11,7%) | 12 (9,4%) | 13 (14,6%) | 40 (11,6%) | 0,80 | 0,36 - 1,75 | 0,572 | 0,68 | 0,30 - 1,52 | 0,343 |
| Moderate GCA | 0 | 3 (2,4%) | 5 (5,6%) | 8 (2,3%) | 1,65 | 0,39 - 7,06 | 0,501 | 0,90 | 0,20 - 4,07 | 0,894 |
| Severe GCA | 0 | 0 | 0 | 0 | - | - | - | - | - | - |
| **Specific Atrophy** | 30 (23,4%) | 40 (31,5) | 30 (33,7%) | 100 (29,1%) | 0,56 | 0,32 - 0,99 | 0,044 | 0,53 | 0,30 - 0,94 | 0,030 |
| **White Matter Changes Pons** | 17 (13,3%) | 21 (16,5%) | 17 (19,1%) | 55 (16 %) | 1,75 | 0,95 - 3,22 | 0,074 | 1,84 | 0,98 - 3,45 | 0,057 |
| **Infarction** | 18 (14,1%) | 15 (11,8%) | 14 (15,7%) | 47 (13,6%) | 0,95 | 0,47 - 1,91 | 0,877 | 0,89 | 0,44 - 1,84 | 0,763 |
| **Lacunar infarction** | 12 (9,5%) | 10 (7,8%) | 11 (12,2%) | 33 (9,5%) | 1,03 | 0,46 – 2,32 | 0,936 | 1,00 | 0,44 – 2,27 | 0,993 |
| **CMB Cerebral microbleeds** | 24 (18,8%) | 40 (31,5%) | 27 (30,3%) | 91 (26,3%) | - | - | - | - | - | - |
| Quantity |  |  |  |  |  |  |  |  |  |  |
| 1 microbleed | 12 (9,4%) | 28 (22%) | 16 (18%) | 56 (61,5%) | - | - | - | - | - | - |
| 2-10 microbleeds | 10 (41,7%) | 10 (25%) | 8 (9%) | 28 (30,8%) | - | - | - | - | - | - |
| 10-99 microbleeds | 1 (0,8%) | 0 | 2 (2,2%) | 3 (3,3%) | - | - | - | - | - | - |
| > 100 microbleeds | 1 (0,8%) | 2 (1,6%) | 1 (1,1%) | 4 (4,4%) | - | - | - | - | - | - |
| Location |  |  |  |  |  |  |  |  |  |  |
| lobar | 15 (11,7%) | 22 (17,3%) | 17 (19,1%) | 54 (59,3%) | - | - | - | - | - | - |
| deep | 2 (1,6) | 6 (4,7%) | 2 (2,2%) | 10 (11%) | - | - | - | - | - | - |
| cerebellum | 2 (1,6%) | 5 (3,9%) | 4 (4,5%) | 11 (12,1%) | - | - | - | - | - | - |
| combined lobar/deep | 2 (1,6%) | 2 (1,6%) | 3 (3,4%) | 7 (7,7%) | - | - | - | - | - | - |
| pons/other | 1 (0,8%) | 3 (1,6) | 1 (1,1%) | 5 (5,5%) | - | - | - | - | - | - |
| missing information |  |  |  | 4 (4,4%) |  |  |  |  |  |  |

^A^ Multivariate regression analysis with age and gender as model covariates.

###### Table III

Peripheral blood-pressure measurements are given for the total cohort. Odds ratios (OR) for coexisting CMB are given from univariate and multivariate logistic regression analysis.

|  | Total cohort n=344 | | | | | | |
| --- | --- | --- | --- | --- | --- | --- | --- |
|  | n (%) | Univariate | | | Multivariate^A^ | | |
|  |  | OR | 95% CI | p | OR | 95% CI | p |
| Systolic blood pressure (SBP)  Mean (median), range | 144 (142), 98 - 210 | 1,01 | 1,00 - 1,03 | 0,110 | 1,01 | 1,00 - 1,03 | 0,062 |
| Systolic hypertension | 193 (56,1%) | 1,54 | 0,94 – 2,54 | 0,087 | 1,69 | 1,01 – 2,83 | 0,048 |
| missing | 4 |  |  |  |  |  |  |
| Diastolic blood pressure (DBP)  mean (median), range | 78 (79), 45 -100 | 1,01 | 0,98 - 1,04 | 0,600 | 1,01 | 0,98 - 1,04 | 0,492 |
| Diastolic hypertension | 37 (10,8%) | 2,34 | 1,16 - 4,72 | 0,018 | 2,26 | 1,11 - 4,61 | 0,025 |
| missing | 10 |  |  |  |  |  |  |
| Hypertension (SBP or DBP) | 200 (58,1%) | 1,74 | 1,04 - 2,90 | 0,034 | 1,93 | 1,13 - 3,28 | 0,016 |
| Peripheral Pulse pressure mm Hg left arm  mean (median), range | 65 (62), 25 -128 | 1,01 | 1,00 - 1,03 | 0,121 | 1,01 | 1,00 - 1,03 | 0,083 |
| 0-60 | 157 (45,6%) | 1 | - | - | 1 | - | - |
| 61-80 | 108 (31,4%) | 1,87 | 1,07 - 3,28 | 0,028 | 2,12 | 1,19– 3,78 | 0,011 |
| >81 | 69 (20,1%) | 1,83 | 0,97 - 3,46 | 0,064 | 1,84 | 0,95 - 3,56 | 0,072 |
| Ankle-brachial index (ABI) left side mean (median), range | 1,13 (1,14), 0,51 -1,74 | 1,32 | 0,26 - 6,60 | 0,736 | 0,62 | 0,12 - 3,30 | 0,573 |
| ABI <0,9 | 23 (6,6%) | 1,20 | 0,48 - 3,04 | 0,696 | 1,28 | 0,49 - 3,34 | 0,614 |
| ABI 0,9 – 1,29 | 270 (78,0%) | 1 | - | - | 1 | - | - |
| ABI > 1,3 | 35 (10,1%) | 0,81 | 0,35 - 1,88 | 0,630 | 0,57 | 0,24 - 1,37 | 0,209 |
| Orthostatic intolerance with symptoms at tilting the past year | 106 (30,8%) | 1,23 | 0,74 - 2,05 | 0,435 | 1,22 | 0,72 - 2,05 | 0,465 |
| Orthostatic hypotension at tilting during medical examination | 36 (10,5%) | 1,00 | 0,46 - 2,16 | 0,996 | 0,90 | 0,40 - 2,01 | 0,800 |
| BP-phenotypes: |  |  |  |  |  |  |  |
| Healthy controls^1^ | 72 (20,9%) | 1 | - | - | 1 | - | - |
| Well controlled hypertension^2^ | 63 (18,3%) | 1,70 | 0,74 - 3,94 | 0,215 | 1,49 | 0,63 - 3,54 | 0,368 |
| Ill-controlled hypertension^3^ | 92 (26,7%) | 2,54 | 1,14 - 5,41 | 0,016 | 2,30 | 1,05 - 5,05 | 0,037 |
| Untreated hypertension / WCS^4^ | 90 (26,2%) | 1,82 | 0,84 - 3,95 | 0,131 | 2,02 | 0,91 - 4,51 | 0,085 |
| Previous anti-hypertensive treatment^5^ | 10 (2,9%) | 2,14 | 0,48 - 9,49 | 0,315 | 2,23 | 0,48 - 10,36 | 0,307 |
| Missing/excluded | 17 (4,9%) | - | - | - |  |  |  |
|  |  |  |  |  |  |  |  |
| Duration of anti-hypertensive treatment mean (median), range | 14,2 (12), 2-54 | 1,03 | 0,99 - 1,06 | 0,177 |  |  |  |
| no treatment | 185 (53,8%) | 0,95 | 0,49 - 1,83 | 0,869 | 1,10 | 0,56 - 2,18 | 0,780 |
| 1-10 years | 66 (19,2%) | 1 | - | - | 1 | - | - |
| 11-20 years | 52 (15,1%) | 1,65 | 0,74 - 3,69 | 0,219 | 1,64 | 0,72 - 3,72 | 0,235 |
| >20 years | 28 (8,1%) | 2,02 | 0,79 - 5,20 | 0,144 | 1,99 | 0,76 - 5,23 | 0,162 |
|  |  |  |  |  |  |  |  |

^A^ Multivariate regression analysis with age and gender as model covariates.

^1^  no present or previous anti-hypertensive treatment, no BP lowering drugs, no drugs with indication hypertension, not hypertensive a medical examination

^2^ reports anti-hypertensive treatment, takes BP lowering drugs with indication hypertension, not hypertensive at medical examination

^3^ reports anti-hypertensive treatment, takes BP lowering drugs with indication hypertension, hypertensive at medical examination.

^4^ no present or previous anti-hypertensive treatment, no BP lowering drugs, no drugs with indication hypertension, hypertensive at medical examination. (WCS=white coat syndrome)

^5^ no present anti-hypertensive treatment, reports previous treatment for hypertension, no BP lowering drugs, no drugs with indication hypertension. All cases are hypertensive at medical examination
